# Uso de herramientas de imagenología actual para el estudio del mixoma odontogénico. Una revisión de la literatura

**DOI:** 10.21142/2523-2754-1002-2022-107

**Published:** 2022-06-27

**Authors:** Rocío Vidales-Miranda, Gustavo Adolfo Fiori-Chíncaro, Ana María Agudelo-Botero, Jhoana Mercedes Llaguno-Rubio

**Affiliations:** 1 Universidad Mayor, Real y Pontificia de San Francisco Xavier de Chuquisaca. Sucre, Bolivia. rociovidales8@gmail.com Universidad Mayor de San Francisco Xavier Universidad Mayor, Real y Pontificia de San Francisco Xavier de Chuquisaca Sucre Bolivia rociovidales8@gmail.com; 2 División de Radiología Bucal y Maxilofacial de la Universidad Científica del Sur. Lima, Perú. gfiori@ilaeperu.com, academico@ilaeperu.com Universidad Científica del Sur División de Radiología Bucal y Maxilofacial Universidad Científica del Sur Lima Peru gfiori@ilaeperu.com academico@ilaeperu.com; 3 Universidad Autónoma de Manizales. Manizales, Colombia. ortoamariabotero@gmail.com Universidad Autónoma de Manizales Universidad Autónoma de Manizales Manizales Colombia ortoamariabotero@gmail.com

**Keywords:** mixoma, radiología, tomografía computarizada de haz cónico, myxoma, radiology, cone-beam computed tomography

## Abstract

En la actualidad, el mixoma odontogénico (MO) se clasifica como tumor benigno de origen ectomesenquimal, que se observa entre la segunda y cuarta década de edad, con predilección en mujeres y predominio en región mandibular, compuesto por células redondeadas y angulares incrustadas en un estroma mixoide abundante, de comportamiento biológico agresivo, con la característica de ser localmente infiltrativo; las células tumorales llegan a diseminarse en el espacio intratrabecular más allá del límite óseo. Difícilmente se llega a determinar el borde del tumor, incluso sin continuidad cortical presentando contacto directo con el tejido blando circundante. El aspecto puede ser unilocular (lesiones pequeñas), o comúnmente la imagen típica es la de una lesión destructiva de bordes mal definidos con patrón de crecimiento multilocular, para ello el uso de imágenes médicas avanzadas como la tomografía computarizada de haz cónico (TCHC), la tomografía computarizada (TC) y la resonancia magnética (RM) son de utilidad para establecer un diagnóstico adecuado con la capacidad de señalar las características del MO con precisión y perspectiva tridimensional. Esta revisión presenta un análisis sistematizado sobre los tipos de herramientas imagenológicas que son utilizados actualmente para el estudio del MO.

## Abstract (en)

Odontogenic myxoma (OM) is currently classified as a benign tumor of ectomesenchymal origin, observed between the second and fourth decade of age, with a predilection in women and predominance in the mandibular region, composed of rounded and angular cells embedded in an abundant myxoid stroma, of aggressive biological behavior, having the characteristic of being locally infiltrative. Tumor cells spread in the intratrabecular space beyond the bone limit, making it difficult to determine the edge of the tumor, even without cortical continuity, presenting intermittent direct contact with soft tissue; the appearance can be unilocular (small lesions), or commonly the typical image is that of a destructive lesion with poorly defined borders with a multilocular growth pattern, for which the use of advanced medical images such as Cone Beam Computed Tomography (CTHC), Computed Tomography (CT), Magnetic Resonance Imaging (MRI), are useful to establish an adequate diagnosis with the ability to point out the characteristics of OM with precision and three-dimensional perspective. This review presents a systematic analysis of the types of imaging tools currently used for the study of OM.

## INTRODUCCIÓN

Según la Organización Mundial de la Salud (OMS), el mixoma odontogénico (MO) se clasifica como tumor benigno de origen ectomesenquimal ^1^. Existen dos tipos de mixoma de los maxilares; el mixoma central (intraóseo) y periférico (situado en tejidos blandos) ^2^. Es un tumor benigno, localmente agresivo, no encapsulado, llega a invadir el tejido óseo circundante, aparece entre la segunda y cuarta década de edad, con predilección en mujeres, rara vez se localiza en el maxilar superior, observándose predominio en la región mandibular ^3^^,^^4^.

Generalmente, observamos el MO como hallazgo mediante las radiografías convencionales (RC), pero no es lo bastante fidedigna para mostrar la extensión y las finas estructuras internas del tumor, para ello el uso de imágenes médicas avanzadas como la tomografía computarizada de haz cónico (TCHC), la tomografía computarizada (TC), resonancia magnética (RM), son de utilidad para establecer un diagnóstico adecuado con la capacidad de señalar las características del MO con precisión y perspectivas tridimensionales ^5^.

Las RC presentan características imagenológicas que dificultan un diagnóstico preciso como el desplazamiento y la migración de las piezas dentarias, que se observan con frecuencia y no se perciben en los cortes y planos específicos de los exámenes radiográficos de la TC y la RM, en algunos casos se observa reabsorción radicular de piezas dentales en el área del tumor. Asimismo, las estructuras internas de los tumores muestran diferentes aspectos radiológicos y aparecen como imágenes radiolúcidas uniloculares o multiloculares, con trabéculas finas delicadas o en ocasiones gruesas, que muestran una apariencia de panal de abeja, burbuja de jabón, raqueta de tenis o tela de araña ^5^^-^^7^.

La evaluación con la TCHC es muy útil para señalar las estructuras internas de las lesiones con precisión, evitando la distorsión geométrica y la superposición de estructuras, otorgando información minuciosa para el diagnóstico de MO, por la alta tasa de recurrencia, por eso, la apariencia radiográfica precisa es de gran importancia para llegar a un diagnóstico correcto y a una buena planificación quirúrgica ^5^.

La evaluación de la TC en configuración de la ventana de tejidos blandos, los valores de atenuación del MO se compararon con los músculos circundantes y aparecen como hipodensos y masas isodensas. La TC en configuración de la ventana ósea, revela la extensión del MO a las estructuras circundantes con interrupción de corticales formando lobulaciones y formación de grietas ^6^.

La RM ayuda a detectar intensidades de señal en el tumor y compararlas con las de las estructuras circundantes. Se observa como masas multiloculadas, expansivas con definición más exacta, generalmente con un patrón de crecimiento lobulado, pequeñas grietas o septación parcial, en su composición interna tiene la apariencia de tumores heterogéneos de hipo/isointensidad mixta. Varios autores nombran a la RM dinámica o realce de contraste con el fin de poder diferenciar tumores benignos de malignos y poder diferenciarlos entre los mismos ^6^^,^^8^.

Como otro método de estudio para observar las características imagenológicas del MO, se puede destacar el uso de la tomografía helicoidal multicorte (THM) con administración de contraste endovenoso, la cual permite obtener datos adicionales para la caracterización de la estructura interna de la lesión, diferenciándola así de otras patologías ^9^.

A la fecha, existen pocos trabajos que describen los componentes moleculares del MO, por lo cual se debe establecer un perfil inmunohistoquímico de diversos marcadores tumorales y analizar la microdensidad vascular, con el fin de discutir sus posibles implicaciones en el comportamiento biológico del MO. En este sentido, el propósito de este estudio fue dar a conocer los tipos de herramientas de imagenología actual que son utilizados para el estudio del mixoma odontogénico, mediante la evaluación de imágenes de radiografías convencionales, tomografía computarizada, tomografía computarizada de haz cónico y resonancia magnética. Se analizó también el estudio de uso de la tomografía helicoidal multicorte (THM) con administración de contraste endovenoso comparando las características que muestran y el aporte de cada una de ellas para el diagnóstico del MO de manera rutinaria.

## MATERIALES Y MÉTODOS

Se realizó una búsqueda de la literatura científica en las principales fuentes de información como Medline (vía PubMed), Elsevier, SciELO y Lilacs, usando los términos de búsqueda en el rango de enero de 2012 a diciembre de 2021. Los artículos seleccionados debieron incluir información referente a mixoma, radiología, tomografía computarizada de haz cónico.

## RESULTADOS Y DISCUSIÓN

### Características histopatológicas, fisiológicas y clínicas del mixoma odontogénico

El mixoma odontogénico es una neoplasia benigna, de histogénesis incierta de comportamiento biológico agresivo, teniendo la característica de ser localmente infiltrativo. En 1871, Virchow utilizó el término “mixoma” para describir tumores que histológicamente semejaban el tejido mucinoso del cordón umbilical ^10^. En la actualidad, la Organización Mundial de la Salud lo define como tumores odontogénicos benignos de origen ectomesenquimal, compuesto por células redondeadas y angulares incrustadas en un estroma mixoide abundante ^1^^,^^11^^,^^12^.

 Las células tumorales muestran un núcleo pequeño y picnótico, y suelen mostrar prolongaciones citoplásmicas elongadas que se anastomosan con el citoplasma de las células adyacentes. El estroma mixoide intercelular está compuesto por una matriz rica en mucina (mucopolisacáridos ácidos, fundamentalmente ácido hialurónico y en menor proporción, condroitín sulfato) ^13^^-^^15^.

Las islas de epitelio odontogénico inactivo en ocasiones pueden estar presentes en cantidades mínimas, se cree que su derivación odontogénica está dada por la porción mesenquimal primitiva del germen dental o el ligamento periodontal, que tiene efectos inductores en los nidos del epitelio odontogénico. Puede presentar áreas focales de colágeno y hialinización externa de vasos sanguíneos; en la periferia del tejido mixomatoso se observa invasión dentro de los espacios trabeculares que producen islotes de hueso residual, muestran poca encapsulación histológica y se extienden hacia el tejido blando a través de las estructuras óseas, aunque el borde se observa claramente en el tumor dentro del hueso, las células tumorales llegan a diseminarse en el espacio intratrabecular más allá del límite óseo llegando difícilmente a determinar el borde del tumor, incluso en tumores grandes sin continuidad cortical y con contacto directo con el tejido blando circundante. Esta característica explica la dificultad en la extirpación conservadora de la lesión y el aspecto del borde puede reflejar la naturaleza benigna de este tumor ^6^^,^^12^^,^^16^^,^^17^.

Estas lesiones se caracterizan por conservar células en forma fusiforme o estrelladas en una rica matriz extracelular mixoide con poco colágeno, por lo que aquellos casos en los que se observan grandes cantidades de colágeno se denominan mixofibroma ^18^^-^^20^.

Hay dos formas de mixomas o fibromixomas que se reconocen en la región de la cabeza y el cuello; una se deriva del esqueleto facial llamada también mixoma central y la otra se deriva del tejido blando, que se reconoce como mixoma periférico ^19^^,^^21^.

El mixoma periférico es un tumor odontogénico extremadamente raro, una lesión menos agresiva en comparación con el MO central, de crecimiento lento, presentándose en la encía, macroscópicamente tiene forma de tejido gelatinoso con aspecto histológico de tipo mixoide; la ubicación también es en los maxilares; sin embargo, es poco común y se presenta como una masa submucosa asintomática, usualmente localizada en el paladar ^22^^-^^24^.

Clínicamente el MO central se caracteriza por presentar un aumento progresivo de volumen, con el potencial de causar cambios en el aspecto facial de los pacientes y que se hacen más evidentes cuando la lesión alcanza dimensiones importantes que interfieren con la oclusión, con desplazamientos dentales que llevan a presentar alteraciones como úlceras de los tejidos subyacentes ^25^.

Algunos autores reportan casos en la mandíbula en la que se presenta parestesia, esta última es un signo clínico bastante raro, que puede llegar a producir el desplazamiento del nervio alveolar inferior como consecuencia de una compresión directa de la lesión hacia estructuras anatómicas adyacentes, presenta reabsorción radicular y expansión de corticales como signo clínico asociado a esta neoplasia ^4^^,^^25^.

Estas características hacen que los tratamientos del MO varíen desde la enucleación simple y el legrado hasta la resección segmentaria y la hemimandibulectomía. Las tasas de recurrencia son altas, alrededor del 25%, especialmente cuando se adopta un enfoque más conservador, las recurrencias se presentan secundarias a márgenes inadecuados, dado la naturaleza gelatinosa o mucoide y la falta de cápsula en la mayoría de los casos que se presenta el MO. Actualmente, no existen pautas claras de manejo quirúrgico basadas en la evidencia para el mixoma odontogénico ^15^^,^^26^^,^^27^.

### Características imagenológicas que presenta el mixoma odontogénico en estudios y diagnósticos diferenciales con el uso de estudios 2D y 3D

Según las diferentes cantidades de componente mixoide y tejido fibroso, y el grado de polarización celular, el MO puede mostrar una apariencia radiográfica más compleja de lo que se conoce (Tabla 1). El aspecto puede ser unilocular (lesiones pequeñas) o, comúnmente, la imagen típica es la de una lesión destructiva de bordes mal definidos con patrón de crecimiento multilocular con preferencia por la región posterior de la mandíbula ^21^^,^^28^^,^^29^.

**Tabla 1:** Aspectos imagenológicos del mixoma odontogénico en radiografías panorámicas

	Locularidad	Patrón óseo	Tamaño
TIPO I	Radiolucidez Unilocular	Bordes definidos, densidad interna, sin trabeculación interna.	Menos de 2 cm
TIPO II	Multilocular, área radiolúcida con algunas hebras de trabéculas intratumorales delicadas o gruesas	Expansión con margen visible	Más de 2 cm
TIPO III	Radiolucidez con trabeculación recta y angular ^(^apariencia de raqueta de tenis)	Destrucción cortical ^(^perforación)	Entre 3 y 5 cm
TIPO IV	Radiolucidez con compartimientos redondos u ovales formados por trabeculación curva ^(^apariencia de panal)	Destrucción cortical con extensión fuera del hueso. Este aspecto característico puede usarse para distinguir esta lesión del ameloblastoma y el carcinoma gingival.	Más de 5 cm
TIPO V	Combinación de 2 o más de los anteriores, radiográficamente muestra una imagen “carcomida” en el margen de la lesión.	Bordes mal definidos y difusos, lo que causa desplazamiento de piezas dentarias.	Más de 5 cm
TIPO VI	Destrucción osteolítica y osteogénesis, lo que da la apariencia reticulada o la apariencia de “rayos de sol”.	Bordes mal definidos, apariencia que puede llegarse a confundir con características imagenológicas del carcinoma gingival u otros tumores malignos.	Tamaño más grande, causando deformidad facial

Las características de las imágenes de la TCHC muestran una alta resolución espacial, expansión del MO, interrupción del margen cortical, septos finos y rectos que se reconocen para separar el tumor en espacios triangulares, cuadrados o rectangulares, tabiques que, frecuentemente, se dispersan a los bordes de las lesiones y aparecen perpendiculares a los márgenes. Se muestra una tendencia a involucrar el proceso alveolar, festonear las raíces y afectar la integridad de la cresta alveolar ^6^^,^^11^

El estudio con la TC señala características como multilocularidad con septos óseos definidos; en cambio, las lesiones uniloculares mostraron algunas trabéculas que se perciben, principalmente, hacia la periferia del tumor, acompañado de filamentos de trabéculas de densidad fina parecida a un cordón. Algunos autores afirman que la presencia o ausencia de loculación describe la etapa de desarrollo del tumor ^5^^,^^8^.

Con la precisión de la TC se demuestra la interrupción de la cortical en áreas localizadas, observándose, aun así, la delimitación del tejido circundante de los tumores, el periostio procede como una barrera y evita la extensión de la lesión hacia el tejido blando en algunos casos, esto causa la compresión del tejido blando, que puede llegar a comportarse como una pseudocápsula ^5^.

Morgan señala que la densidad de los tumores en comparación con los músculos, se mide a través de las unidades Hounsfield en TC. Las unidades Hounsfield se definen como transformaciones lineales de los coeficientes de atenuación de rayos X medidos de un material con referencia al agua; en la escala HU, el aire tiene un valor de -1000 HU, el agua tiene un valor de 0 HU y el hueso denso tiene un valor de +1000 HU, como valores aproximados las densidades del MO presentan una consistencia espesa, pero acuosa (HN para agua: 0, para CSF: 15), hasta un tejido que es más denso que el músculo (HN: 50) y la médula ósea (HN: 300) ^6^^,^^30^.

En la RM se perciben las paredes de los tumores y los patrones de crecimiento nítidamente representados, el MO muestra una pared lisa; no obstante, en las áreas focales se observa borde festoneado, grietas o lobulaciones, estas características apoyan la naturaleza infiltrativa o invasiva del MO ^6^^,^^8^.

El MO tiene una apariencia radiográfica variable y desafiante porque sus características se superponen con las de otras neoplasias benignas y malignas, incluyendo ameloblastoma, granuloma central de células gigantes, hemangioma intraóseo, quistes óseos aneurismáticos y osteosarcoma, que a menudo conducen a un diagnóstico erróneo (Tabla 2) ^15^^,^^29^^,^^31^.

**Tabla 2:** Aspectos comparativos para el diagnóstico diferencial del mixoma odontogénico

Diagnóstico diferencial	Mixoma	Ameloblastoma	Granuloma de células gigantes	Osteosarcoma
Definición	Es una neoplasia benigna, de histogénesis incierta de comportamiento biológico agresivo, con la característica de ser localmente infiltrativo.	La OMS lo define como una neoplasia polimórfica localmente invasiva que comúnmente tiene un patrón folicular o plexiforme, en un estroma fibroso.	Reacción de reparación local debido posiblemente a una hemorragia o a un traumatismo, o mejor, un análogo al verdadero tumor de células gigantes de los huesos largos.	Tumor maligno de tipo mesenquimático cuyas células cancerígenas producen una matriz osteoide
Origen	Origen ectomesenquimal	De los componentes epiteliales del desarrollo del diente como: restos de la lámina dental, epitelio reducido del esmalte, restos de Malassez y capas de células basales del epitelio superficial suprayacente odontogénico benigno	Lesión proliferativa no neoplásica de etiología desconocida	Origen mesenquimático
Histología	Compuesto por células redondeadas y angulares incrustadas en un estroma mixoide abundante	Presencia de células eosinofílicas, llamadas paracaídas o paraguas en la superficie luminal	Tejido fibroso, se observa múltiples focos hemorrágicos, acumulación de células gigantes multinucleadas, en un estroma que contiene células mesenquimales de forma ovoide y trabéculas de hueso inmaduro.	Tejido sarcomatoso altamente maligno, en donde las células producen directamente hueso neoplásico o sustancia osteoide, sin interposición de osteoblastos normales.
Localización	Zona posterior mandibular	Región posterior del maxilar inferior	Mandíbula, línea media y zona de premolares	Región molar del maxilar inferior
Edad	2.a-4.a década de vida	3.a-4.a década	3.a década de vida	3.a década de vida
Sexo	Femenino	Femenino	Femenino	Ambos sexos
Aspecto radiográfico	El aspecto puede ser unilocular (lesiones pequeñas), o la imagen típica es la de una lesión destructiva de bordes mal definidos con patrón de crecimiento multilocular	Lesiones uniloculares y multiloculares (como “Pompas jabón” si los lóculos son grandes o panal de abeja si son lóculos pequeños). Los dientes del área pueden presentar movilidad por reabsorción radicular a la presión del tumor (reabsorción en filo de cuchilla)	La imagen radiológica no es patognomónica. Puede presentarse de forma unilocular o multilocular y mostrar expansión variable y destrucción de la cortical	Lesión osteolítica, en etapas avanzadas puede manifestarse como lesiones radiolúcidas moteadas o radiopacidades irregulares mal delimitadas (mixtas) y en algunos casos se aprecia radiopacidad característica en forma de rayo de sol debido a la reacción perióstica.

Los compartimientos de un ameloblastoma son redondos en lugar de los espacios cuadrados o triangulares de los MO, el margen y el límite cortical son más delimitados, con menor posibilidad que invada los tejidos blandos o afecte la continuidad cortical. Luo *et al.*^32^ informaron que el ameloblastoma desmoplásico presentaba la apariencia de panal, presentando desplazamiento de la raíz, parecido a los MO; sin embargo, el ameloblastoma generalmente compromete las regiones anterior y premolar del maxilar inferior y tiene una alta tendencia a expandirse en el lado bucal ^5^^,^^31^^-^^33^.

Los granulomas centrales de células gigantes generalmente se presentan en pacientes menores de 30 años y con predilección en la zona anterior del maxilar superior e inferior, presenta características como una probable expansión, que conduce al adelgazamiento de la corteza ^34^.

El hemangioma intraóseo generalmente afecta el conducto dentario inferior provocando un ensanchamiento anormal del canal mandibular, el agujero mentoniano y el agujero mandibular. Clínicamente, puede presentar enrojecimiento gingival y hemorragia. Es necesario un estudio histopatológico y el uso de herramientas imagenológicas para confirmar un diagnóstico preciso de MO ^5^^,^^28^.

Dabbaghi *et al*. ^16^ y otros autores describen la perforación de la placa cortical y la línea hiperdensa extendiéndose desde el periostio hacia el tejido blando, dando forma de “rayos solares” una característica rara del MO, estas características sugieren la naturaleza invasiva del tumor, por lo que se aconseja diferenciarla radiográficamente de las lesiones metastásicas en la mandíbula y el sarcoma osteogénico ^5^^,^^16^^,^^35^^-^^37^.

### Cualidades de las diferentes herramientas imagenológicas y propuesta de nuevas alternativas para el análisis del MO

La apariencia radiográfica del MO no es específica, varía considerablemente; por lo tanto, para establecer un diagnóstico adecuado y brindar información detallada sobre la extensión del tumor, el borde, la forma y el compromiso con las estructuras circundantes, se necesita el uso de estudio radiográficos adecuados para un diagnóstico y tratamiento correcto.

El examen imagenológico básico para una lesión del MO inicia con una radiografía panorámica, considerando los efectos de distorsión de este tipo de estudio y que no ofrece una calidad suficiente para un análisis preciso y detallado de la textura del hueso. Las herramientas imagenológicas más avanzadas como la tomografía computarizada y la resonancia magnética pueden ofrecer opciones de diagnóstico, que dan solución a algunas limitaciones de las imágenes 2D ^6^.

El MO por ser un tumor óseo benigno infiltrativo presente en la mayoría de los casos en el esqueleto facial, demuestra diferentes patrones que dificultan el diagnóstico, las cuales no se pueden evaluar en una radiografía panorámica. La TCHC puede evitar la distorsión geométrica, la superposición de estructuras anatómicas y mostrar la estructura interna fina de las lesiones con precisión y su relación con las estructuras circundantes ^5^. En los estudios con TC se llega a observar el margen del tumor, brindando la ventaja de reproducirla en 3D para valorar la verdadera extensión de la lesión, con el fin de poder utilizarlo para la planificación de tratamientos reconstructivos ^6^^,^^16^.

La RM muestra la extensión, el contenido y el patrón de crecimiento del tumor; tiene la ventaja de diferenciar las partes mixomatosas y colágenas dentro del tumor al asignar diferentes intensidades de señal a diferentes tejidos, otorga la fidelidad para determinar la naturaleza vascular del tumor. La diferencia en el patrón de captación de contraste refleja una composición diferente de la masa, que es principalmente colágena en la periferia y mixomatosa hacia el área central ^38^^,^^39^.

La inmunomarcación es un paso secundario y complementario, que en determinadas circunstancias se convierte en el instrumento de definición del diagnóstico, lo cual tiene como consecuencia un tratamiento y pronóstico que impacta directamente en los pacientes. En el MO la reacción inmunohistoquímica de las células neoplásicas a diversos anticuerpos es variable. Frecuentemente, se detecta positividad para marcadores mesenquimales como vimentina, actina músculo-específica y en menor número de casos a proteína S-100; sin embargo, existen nidos epiteliales, en ellos se demuestra reacción positiva a CK19, marcador inespecífico presente en el epitelio odontogénico normal ^14^, así como los marcadores CK19 y CD138 son útiles para detectar epitelio odontogénico en el MO, y pueden ser importantes para el alto potencial invasivo ^40^^,^^41^.

## CONCLUSIONES

El mixoma odontogénico (MO) es un tumor benigno que suele ser evidenciado como un hallazgo imagenológico, generalmente a través de estudios por imágenes de rutina 2D; sin embargo, estas imágenes no son capaces de mostrar detalles como la configuración interna y externa de la lesión, así como su extensión ni el compromiso de las estructuras anatómicas adyacentes. Por ello, en la actualidad se aplican otros estudios imagenológicos, algunos con radiación ionizante como la tomografía axial computarizada (TAC), la tomografía computarizada de haz cónico (TCHC) o la tomografía axial computarizada helicoidal o multidetector (THM), que permiten evaluar las lesiones con mayor precisión, gracias a que evitan la distorsión geométrica y la superposición de estructuras anatómicas. Así mismo, pueden usarse imágenes no ionizantes como las obtenidas con resonancia magnética, que permite, además, evaluar el componente blando de la lesión y su relación con estructuras adyacentes.

## Fuente de financiamiento:

Autofinanciado
